# Glycosaminoglycan signatures in body fluids of mucopolysaccharidosis type II mouse model under long-term enzyme replacement therapy

**DOI:** 10.1007/s00109-022-02221-3

**Published:** 2022-07-11

**Authors:** Francesca Maccari, Laura Rigon, Veronica Mantovani, Fabio Galeotti, Marika Salvalaio, Francesca D’Avanzo, Alessandra Zanetti, Federica Capitani, Orazio Gabrielli, Rosella Tomanin, Nicola Volpi

**Affiliations:** 1 Department of Life Sciences, University of Modena and Reggio Emilia, Via Campi 213/D, 41125 Modena, Italy; 2 Department of Women’s and Children’s Health, University of Padova, Via Giustiniani 3, 35128 Padua, Italy; 3 Fondazione Istituto di Ricerca Pediatrica Città Della Speranza, Padua, Italy; 4 Clinical and Experimental Medicine PhD Program, University of Modena and Reggio Emilia, Modena, Italy; 5 Department of Pharmaceutical and Pharmacological Sciences, University of Padova, Padua, Italy; 6 Department of Clinical Sciences, Division of Pediatrics, Polytechnic University of Marche, Ospedali Riuniti, Presidio Salesi, Ancona, Italy

**Keywords:** Mucopolysaccharidosis type II, Glycosaminoglycans, Enzyme replacement therapy, Iduronate 2-sulfatase, Heparan-sulfate, Dermatan-sulfate

## Abstract

**Abstract:**

Mucopolysaccharidosis type II (MPS II) is a neurometabolic disorder, due to the deficit of the lysosomal hydrolase iduronate 2-sulfatase (IDS). This leads to a severe clinical condition caused by a multi-organ accumulation of the glycosaminoglycans (GAGs/GAG) heparan- and dermatan-sulfate, whose elevated levels can be detected in body fluids. Since 2006, enzyme replacement therapy (ERT) has been clinically applied, showing efficacy in some peripheral districts. In addition to clinical monitoring, GAG dosage has been commonly used to evaluate ERT efficacy. However, a strict long-term monitoring of GAG content and composition in body fluids has been rarely performed. Here, we report the characterization of plasma and urine GAGs in *Ids* knock-out (Ids-ko) compared to wild-type (WT) mice, and their changes along a 24-week follow-up, with and without ERT. The concentration of heparan-sulfate (HS), chondroitin-sulfate (CS), and dermatan-sulfate (DS), and of the non-sulfated hyaluronic acid (HA), together with their differentially sulfated species, was quantified by capillary electrophoresis with laser-induced fluorescence. In untreated Ids-ko mice, HS and CS + DS were noticeably increased at all time points, while during ERT follow-up, a substantial decrease was evidenced for HS and, to a minor extent, for CS + DS. Moreover, several structural parameters were altered in untreated ko mice and reduced after ERT, however without reaching physiological values. Among these, disaccharide B and HS 2s disaccharide showed to be the most interesting candidates as biomarkers for MPS II. GAG chemical signature here defined provides potential biomarkers useful for an early diagnosis of MPS II, a more accurate follow-up of ERT, and efficacy evaluations of newly proposed therapies.

****Key messages**:**

- Plasmatic and urinary GAGs are useful markers for MPS II early diagnosis and prognosis.
- CE-LIF allows GAG structural analysis and the quantification of 17 different disaccharides.
- Most GAG species increase and many structural features are altered in MPS II mouse model.
- GAG alterations tend to restore to wild-type levels following ERT administration.
- CS+DS/HS ratio, % 2,4dis CS+DS, and % HS 2s are potential markers for MPS II pathology and ERT efficacy.

**Supplementary Information:**

The online version contains supplementary material available at 10.1007/s00109-022-02221-3.

## Introduction

Mucopolysaccharidosis type II (MPS II, Hunter syndrome) is a rare metabolic genetic disorder due to the deficit of the lysosomal enzyme iduronate 2-sulfatase (I2S or IDS) (EC 3.1.6.13), which normally hydrolyzes the 2-sulfate groups of the L-iduronate 2-sulfate units of the glycosaminoglycans (GAGs) heparan-sulfate (HS), dermatan-sulfate (DS), and heparin [1]. The enzyme deficit causes storage of DS and HS, progressively damaging most organs including the brain [2]. Hunter syndrome is the only X-linked MPS, thus primarily affecting males. Babies at birth are normal, but signs and symptoms usually appear in early childhood (2–4 years). The clinical picture varies from attenuated to severe forms and life expectancy can be of 10–20 years and up to 60, with cardiac-respiratory failure being the most common cause of death [2].

Currently approved therapies for the disease include the hematopoietic stem cell transplantation and, since 2006, the enzyme replacement therapy (ERT) [3], although the former has rarely resulted successful in these patients [4]. Systematic review of the first 10 years of treatment evidenced that weekly ERT infusions generally reduce urine GAG levels and liver/spleen volume in MPS II patients, while the effectiveness on other outcomes is variable [5]. Moreover, ERT is unable to help the neurological disease, due to the inability of the recombinant enzyme to cross the blood–brain barrier. However, in the last years, several approaches have been tested to deliver the enzyme to the central nervous system such as intracerebroventricular and intrathecal administration as well as the use of brain-targeted fusion proteins exploiting the concept of the molecular Trojan Horse [2, 6, 7].

Nowadays, the knowledge of MPS pathophysiology is progressing beyond lysosomal and extracellular GAG accumulation, towards the involvement of other signaling pathways [8, 9] and a new view of the lysosome as a regulatory hub for cellular and organismal homeostasis [10]. As for MPS II, the availability of mouse models in the last 20 years, obtained by gene disruption [11–13], has importantly helped to understand the disease pathogenesis and progression [14, 15]. It also revealed to be very useful to monitor the efficacy of experimental therapies [14, 16–22] and more specifically of ERT [23–25].

GAGs are macromolecules constituted by sequences of disaccharides, each of them formed by uronic acid (apart from the keratan-sulfate), glucuronic acid (GlcA), or iduronic acid (IdoA), and an amino sugar, *N*-acetyl-galactosamine (GalNAc) or *N*-acetylglucosamine (GlcNAc) [26]. The only non-sulfated GAG is hyaluronic acid (HA), while chondroitin-sulfate (CS), dermatan-sulfate (DS), and heparan-sulfate (HS) are sulfated to a different extent and in different positions, resulting very heterogeneous for charge density and chemical structure [27, 28]. GAGs are covalently linked to specific proteins to form proteoglycans (PGs), which are located on cell surface, in pericellular regions and in the extracellular matrix, and are involved in a large variety of cellular processes [29]. PGs are characterized by a high structural variability, mainly due to the structural heterogeneity of the GAG chains that seem to be responsible of the PG biological function [30].

Urine GAGs are commonly used as diagnostic biomarkers in most MPSs, as well as markers to monitor ERT efficacy in human recombinant IDS (hr-IDS) clinical trials [31–33]. Already in 1975, Erickson et al. showed that urinary excretion of GAGs was independent of the serum concentration and suggested that the urine GAGs appear as a result of epithelial cell turnover [34]. Although it is still debated if the quantity and composition of urine GAGs faithfully represent the total body disease burden or if they only reflect the renal disease, actually they are still being commonly measured as urine specimens are easily collected and analyzed [35].

Also the analysis of plasma GAGs, even though requiring a more invasive procedure, seems to be very informative, probably reflecting the storage pattern of GAGs in other organs, such as the liver and brain [36]. Indeed, a recent study in MPS I mouse model showed that the oligosaccharide storage pattern in urine reflects the one in the kidney, whereas serum closely reflects the HS:DS ratio of the brain and liver [36]. Thus, the combined analysis of GAGs in both specimens, having different characteristics in terms of storage amount, composition, and sulfation levels, could help to provide information on different aspects of the disease.

With the aim to conduct a punctual, long-term monitoring of ERT administration, we here report GAG characterization in urine and plasma samples of a 24-week follow-up study in Ids-ko mice untreated and treated with hr-IDS, in comparison to wild-type (WT) animals. GAGs were quantitatively and structurally analyzed through capillary electrophoresis with laser-induced fluorescence (CE-LIF) technique [26, 37], a method that quantify a total of 17 variously sulfated disaccharides.

GAG-specific chemical signature as here defined can provide candidate biomarkers of the disease, useful for an early diagnosis, for a more accurate ERT follow-up, and for potential efficacy evaluations of new therapies.

## Materials and methods

### Mouse model

The MPS II mouse model was obtained by the JAX® Mice & Services (JAX stock #024,744; The Jackson Laboratory, Bar Harbor, Maine, USA), originally generated by gene disruption of the murine *Ids* gene [11, 24] and previously characterized [14, 16]. *Ids* knock-out mice (Ids-ko) were expanded in our animal facility in light- and temperature-controlled conditions, with water and food provided ad libitum. This study was conducted in strict accordance with the European Directive 2010/63/EU. The protocol was approved by the Institutional Animal Care and Use Committee of the University of Padua and authorized by the Italian Ministry of Health (project n.410/2015, approved on May 21, 2015).

In the experiments here reported, hemizygous Ids-ko male mice were treated, starting at 12 weeks of age, by weekly administration of Elaprase® (Shire Lexington, MA, USA, now Takeda Pharmaceutical Co.), at a dosage of 1 mg/kg. Administrations were carried out through tail vein injection, for 0, 2, 4, 6, 12, 18, or 24 weeks. As controls, Ids-ko and WT mice were injected with 0.9% NaCl following the same schedule. Before treatment (PRE) and 1 week after the last injection, plasma samples were obtained by submandibular vein bleeding, and urine samples were collected using metabolic cages for 24 h. For each treatment, a group of 6–7 mice was analyzed at each time point.

### Total GAG quantitation by DMB method

Total urine GAG content was determined by DMB (dimethylmethylene blue) method, using the protocol described by de Jong et al. [38] with modifications, as previously reported [18].

### Isolation and purification of GAGs

GAGs were extracted from plasma and urine according to standardized protocols already published [39, 40], including protein digestion, treatment with sodium borohydride and sodium hydroxide to release GAGs from their core proteins, purification on anion-exchange resin, filtration on centrifugal filters having a molecular mass cutoff of 3 kDa, and freeze-drying.

### Structural characterization and quantitation of GAGs

Quantitation and structural characterization of glycosaminoglycans (HA and HS) and galactosaminoglycans (CS and DS) from plasma and urine were performed by constitutive disaccharides. Lyophilized aliquots of isolated GAGs were reconstituted with suitable buffers and treated with chondroitinases or heparinases. Two aliquots of extracted GAGs were reconstituted with ammonium acetate 0.1 M pH 7.9 and treated with 20 milliUnits (mU) of chondroitinase ABC (from *Proteus vulgaris* [EC 4.2.2.4], Sigma-Aldrich) for the determination of HA, CS, and DS, or 20 mU of chondroitinase AC (from *Flavobacterium heparinum* [EC 4.2.2.5], Sigma-Aldrich) at 37 °C overnight for the evaluation of just HA and CS. Another aliquot was reconstituted with 0.1 M sodium acetate and 0.1 M calcium acetate pH 7.0 and treated with a mixture of 1 mU of heparinase I from *Flavobacterium heparinum* [EC 4.2.2.7] and 0.1 mU of heparinases II (from *Flavobacterium heparinum *[EC number not assigned]) and III (from *Flavobacterium heparinum* [EC 4.2.2.8]) (Grampian Enzymes**,** Orkney, UK) at 35 °C overnight, for the release of HS disaccharides.

After lyophilization, generated unsaturated disaccharides were fluorotagged with 2-aminoacridone (AMAC) [41] and separated by CE-LIF, as previously reported [37, 39].

The lyophilized mixtures of ∆-disaccharides were reconstituted with a 0.1 M AMAC solution in glacial acetic acid-DMSO and a freshly prepared 1 M sodium cyanoborohydride in water. After centrifugation at 11,000 g for 3 min, derivatization was performed by incubation at 45 °C for 4 h. Finally, 50% v/v DMSO was added to the samples and aliquots taken for CE-LIF analysis.

Disaccharides fluorotagged with AMAC were analyzed using an HPCE system (Agilent Technologies, Wilmington, DE, USA) equipped with a ZETALIF (Picometrics, France) detector (*λ*_ex_ = 488 nm). Resolution and analysis were performed on uncoated fused-silica capillary columns at 25 °C using 50 mM phosphate buffer (pH 3.8) with a voltage of 30 kV under reversed polarity. Between each run, the capillary was flushed with HPLC-grade water, 0.1 M NaOH, water, and operating buffer. The operating buffer was filtered through a 0.22 μm membrane filter. Samples were introduced using the pressure mode (50 mbar × 5 s) at the cathode.

The single HA, HS and galactosaminoglycans, CS and DS, contents were calculated against real standards (Sigma-Aldrich) enzymatically treated as reported above. The absolute content of different GAGs in plasma was expressed as µg/ml, while in urine, the values were normalized to creatinine (CR) and reported as µg/mg CR. From these concentrations, the ratio (CS + DS)/HS was calculated. Moreover, the relative percentages of unsaturated disaccharides of HS (eight disaccharides) and galactosaminoglycans (eight disaccharides) were measured (Table 1) [26]. Starting from these data, the following parameters were calculated: the ratio between the % of disaccharide 4s and the % of non-sulfated disaccharide (4s/0s) and the ratio between the content of disaccharide sulfated in position six of GalNAc and the percentage of non-sulfated disaccharide (6s/0s). The charge density of sulfated GAGs (galactosaminoglycans and HS) was also calculated considering the presence and the percentages of sulfated groups for each kind of disaccharide. Finally, specific structural features of HS were determined as the percentage of *N*-acetyl, *N*-sulfated, 2-sulfated, and 6-sulfated groups. On the whole, along with the absolute content of the various GAGs, a total of further 9 structural features (CS + DS and HS charge densities; CS + DS/HS ratio; 4s/0s and 6s/0s CS + DS ratios; *N*-acetyl, *N*-sulfated, 2-sulfated, and 6-sulfated HS groups) were measured for both plasma and urine. Minor uncommon HS disaccharides mainly formed of GlcNH or sulfate group in position 3 of the uronic acid were not observed in our CE-LIF analysis as well as unidentified peaks possibly belonging to other HS species.

**Table 1:** Unsaturated disaccharides of chondroitin- (CS), dermatan- (DS), and heparan-sulfate (HS)

***Glycosaminoglycan***	***Disaccharide***
CS + DS	0s: ΔUA-GalNAc
	2s: ∆UA2S-GalNAc
	6s: ∆UA-GalNAc6S
	4s: ∆UA-GalNAc4S
	2,6dis: ∆UA2S-GalNAc6S
	4,6dis: ΔUA-GalNAc4S6S
	2,4dis: ΔUA2S-GalNAc4S
	2,4,6tris: ΔUA2S-GalNAc4S6S
HS	0s: ΔUA-GlcNAc
	Ns: ΔUA-GlcNS
	6s: ΔUA-GlcNAc6S
	2s: ΔUA2S-GlcNAc
	N,6dis: ΔUA-GlcNS6S
	N,2dis: ΔUA2S-GlcNS
	2,6dis: ΔUA2S-GlcNAc6S
	N,2,6tris: ΔUA2S-GlcNS6S

### Statistical analysis

For both DMB and CE-LIF data analysis, statistically significant differences between Ids-ko vs WT and Ids-ko vs treated Ids-ko mice groups were determined by applying the non-parametric Mann–Whitney *U* test using GraphPad Prism 9.3.1 (La Jolla, CA, USA). Significance was set at *p* < 0.05. Data are presented as mean ± standard deviation. All DMB biochemical analyses were repeated at least 3 times.

## Results

At 12 weeks of age, plasma and urine samples were collected from hemizygous Ids-ko and paired WT male mice, for the quantitative and structural characterization of GAGs at the beginning of the study (*T* = 0). GAG evaluation was then performed after 2, 4, 6, 12, 18, and 24 weeks of treatment with Elaprase® or 0.9% NaCl for the controls.

### Quantitation of urine total GAGs by DMB method

**Fig. 1:**
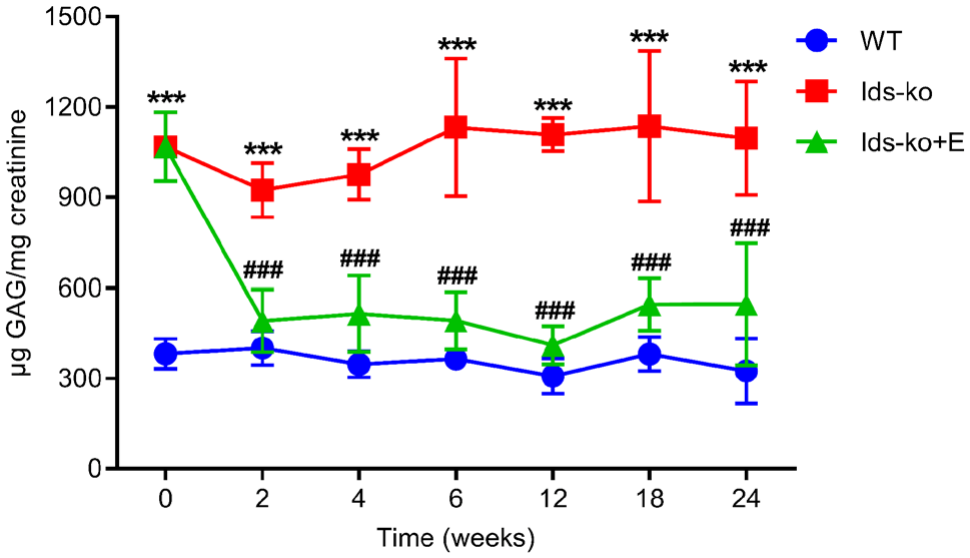
Total urine GAG content by DMB method. Total urine GAGs measured by DMB method in WT, Ids-ko, and ERT-treated Ids-ko (Ids-ko + E) mice, during a time course of 24 weeks at *T* = 0, 2, 4, 6, 12, 18, and 24 weeks. *n* = 6–7 per group. All values are represented as mean ± SD. Asterisks indicate a statistically significant difference between Ids-ko and WT at the same time point (Mann–Whitney *U* test, ***p-value < 0.001); hash marks indicate a statistically significant difference between Ids-ko and Ids-ko + E at the same time point (Mann–Whitney *U* test, ###*p* < 0.001)

Quantitation of total urine GAGs by DMB evidenced, as expected, a statistically significant increase in untreated Ids-ko vs WT mice at all time points. Moreover, total urine GAGs in treated ko-mice (Ids-ko + E) significantly reduced starting from 2 weeks of ERT until the end of the follow-up, and tended to normalize to WT values (Fig. 1).

### Quantitation of plasma and urine HS, galactosaminoglycans, and HA by CE-LIF

**Fig. 2:**
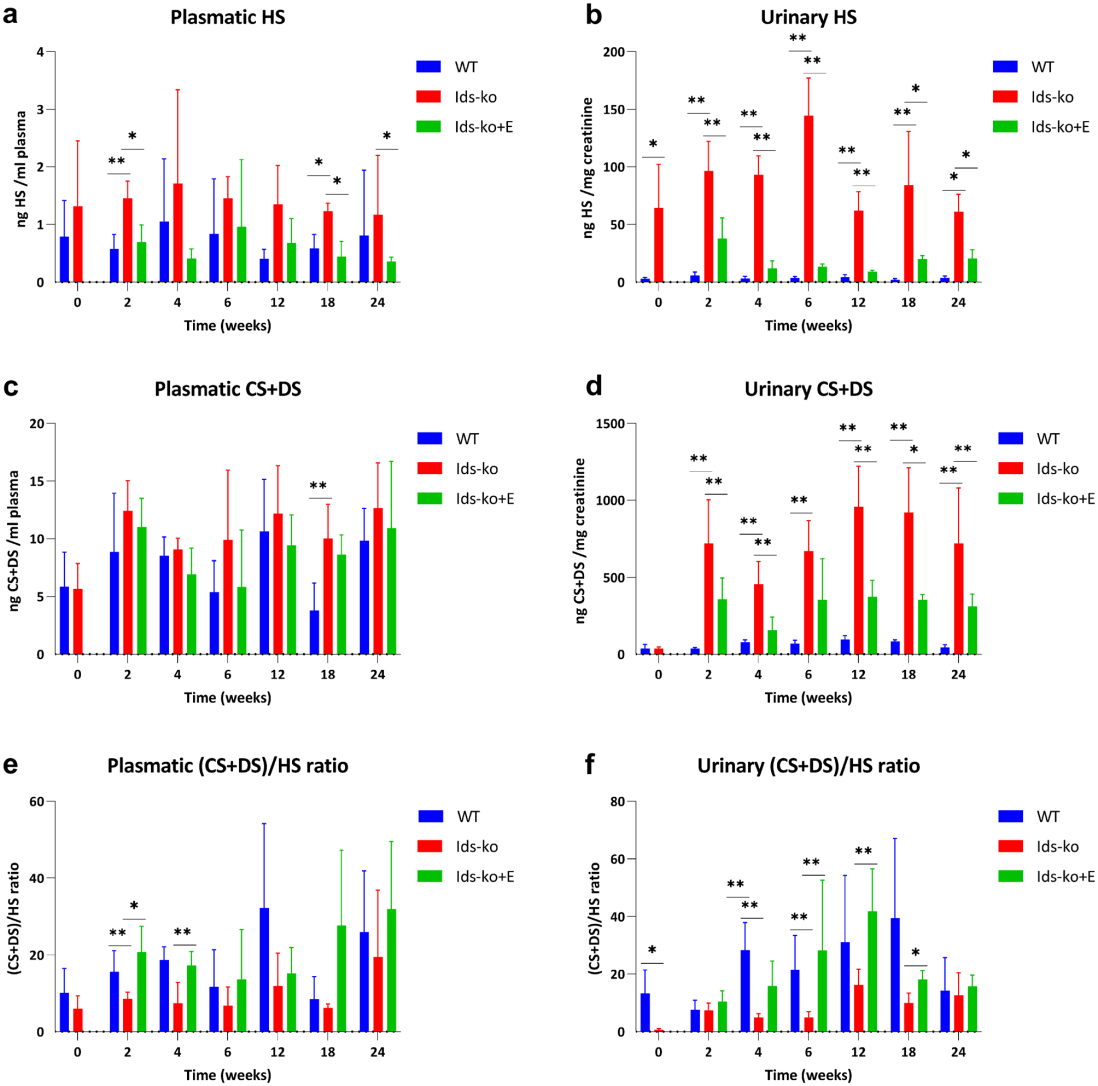
Quantitation of plasma and urine HS, galactosaminoglycans, and galactosaminoglycans/HS ratio by CE-LIF. HS (**a**, **b**), galactosaminoglycans CD + DS (**c**, **d**), and galactosaminoglycans/HS ratio (**e**, **f**) measured in plasma and urine of WT, Ids-ko, and ERT-treated Ids-ko (Ids-ko + E) mice, during a time course of 24 weeks at *T* = 0, 2, 4, 6, 12, 18, and 24 weeks. All values are represented as mean ± SD. *n* = 6–7 per group. Asterisks indicate a statistically significant difference between Ids-ko and WT and between Ids-ko and Ids-ko + E at the same time point (Mann–Whitney *U* test; * 0.01 ≤ *p*-value < 0.05; ** 0.001 ≤ *p*-value < 0.01; *** *p*-value < 0.001)

Quantitation of HS and galactosaminoglycans (CS + DS) in plasma and urine by CE-LIF (Fig. 2a–d) evidenced a variable increase in Ids-ko mouse samples compared to WT ones at all time points. The variations of HS and galactosaminoglycans were more marked in urine with respect to plasma, with HS showing a dramatic increase up to 40-fold at 18 weeks of follow-up. HS variations of ko-mice vs WT were significant at all time points in urine and at 2 and 18 weeks in plasma, while the climb of CS + DS was significant at all time points with exception of *T* = 0 in urine, and only at 18 weeks in plasma. Analogously, the effect of ERT treatment was more visible in urine than in plasma with higher percent variations in HS values with respect to CS + DS ones (− 90% urine HS at 6 weeks). Indeed, hr-IDS treatment caused a significant decline of HS in urine at all time points and in plasma at 2, 18, and 24 weeks (Fig. 2a, b); also galactosaminoglycans varied significantly in urine at 2, 4, 12, 18, and 24 weeks, while no significant variation was detected in plasma (Fig. 2c, d).

A moderate growth in both biological fluids of the non-sulfated HA was also observed in Ids-ko mice, both in plasma (at 4, 6, 18, and 24 weeks) and in urine (at 2, 12, 18 weeks). However, the effect of ERT on HA resulted statistically significant only at 4 weeks in plasma and at 18 weeks in urine (Online Resource 1–Fig. S1).

Being CS + DS substantially more abundant than HS in both plasma (about 10-fold) and urine (about 20-fold), the CS + DS/HS ratio allows to easily evidence the fluctuation of HS with respect to that of galactosaminoglycans. CS + DS/HS drop in Ids-ko vs WT mice was generally more marked in urine than in plasma (Fig. 2e, f); moreover, this variation was statistically significant at 0, 4, and 6 weeks in urine (Fig. 2f) and only at 2 weeks of follow-up in plasma (Fig. 2e). Following treatment, CS + DS/HS ratio tended to restore to physiological values in both biological fluids, with statistically significant reduction from 4 to 18 weeks in urine and at 2 and 4 weeks in plasma (Fig. 2e, f).

### Structural disaccharides analysis of galactosaminoglycans in plasma and urine by CE-LIF

**Fig. 3:**
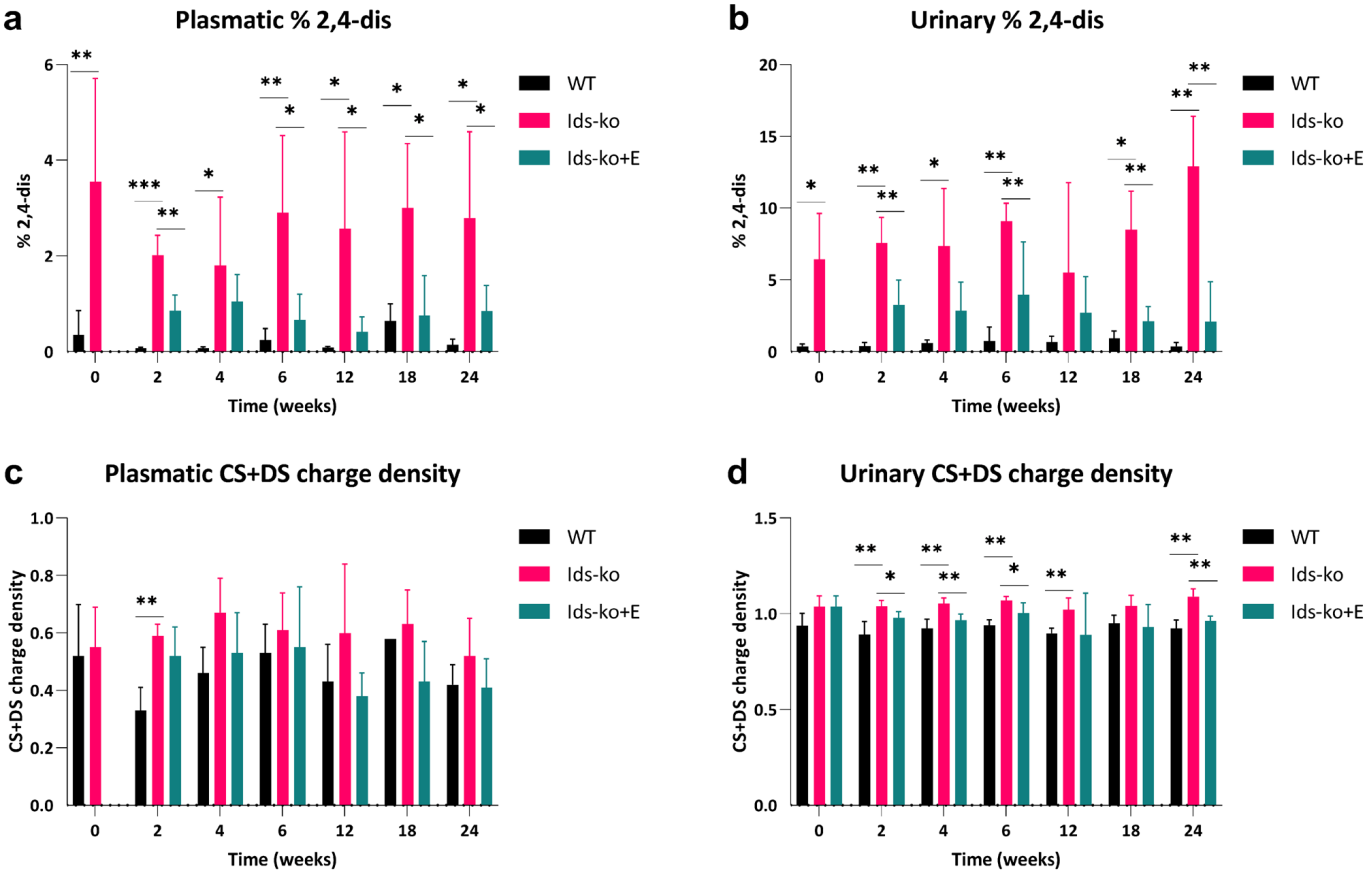
Structural analysis of galactosaminoglycans in plasma and urine by CE-LIF. Percentage content of the disaccharide disulfated in position 2 of uronic acid and 4 of *N*-acetyl-galactosamine (2,4dis) (**a**, **b**) and CS + DS charge density (**c**, **d**) measured in plasma and urine of WT, Ids-ko, and ERT-treated Ids-ko (Ids-ko + E) mice, during a time course of 24 weeks at *T* = 0, 2, 4, 6, 12, 18, and 24 weeks. All values are represented as mean ± SD. *n* = 6–7 per group. Asterisks indicate a statistically significant difference between Ids-ko and WT and between Ids-ko and Ids-ko + E at the same time point (Mann–Whitney *U* test; * 0.01 ≤ *p*-value < 0.05; ** 0.001 ≤ *p*-value < 0.01; *** *p*-value < 0.001)

Structural analysis of galactosaminoglycans evaluated 8 differentially sulfated species both in plasma and urine (Table 1). Among them, we report in Fig. 3a, b the percentage of disaccharide disulfated in position 2 of uronic acid and 4 of GalNAc (disaccharide B or 2,4dis). This percentage showed a statistically significant increase in Ids-ko mice compared to WT at all time points in plasma (Fig. 3a), as well as in urine except at 12 weeks (Fig. 3b), reaching peaks of about 19-fold increase in urine at 2 weeks and of more than 2-fold in plasma at 18 weeks. Moreover, it significantly declined with therapy in plasma at most weeks and in urine at 2, 6, 18, and 24 weeks, with a similar percent decrease in both body fluids (Fig. 3a, b). The analysis CS + DS charge density evidenced notable variations mostly in urine; the increases in the affected animals compared to WT at 2, 4, 6, 12, and 24 weeks, and the reductions in treated mice vs untreated at 2, 4, 6 and 24 weeks of ERT, resulted statistically significant (Fig. 3c, d). The remaining parameter 4s/0s ratio (Online Resource 1–Fig. S2) did not present noteworthy variations.

### Structural disaccharides analysis of HS in plasma and urine by CE-LIF

**Fig. 4:**
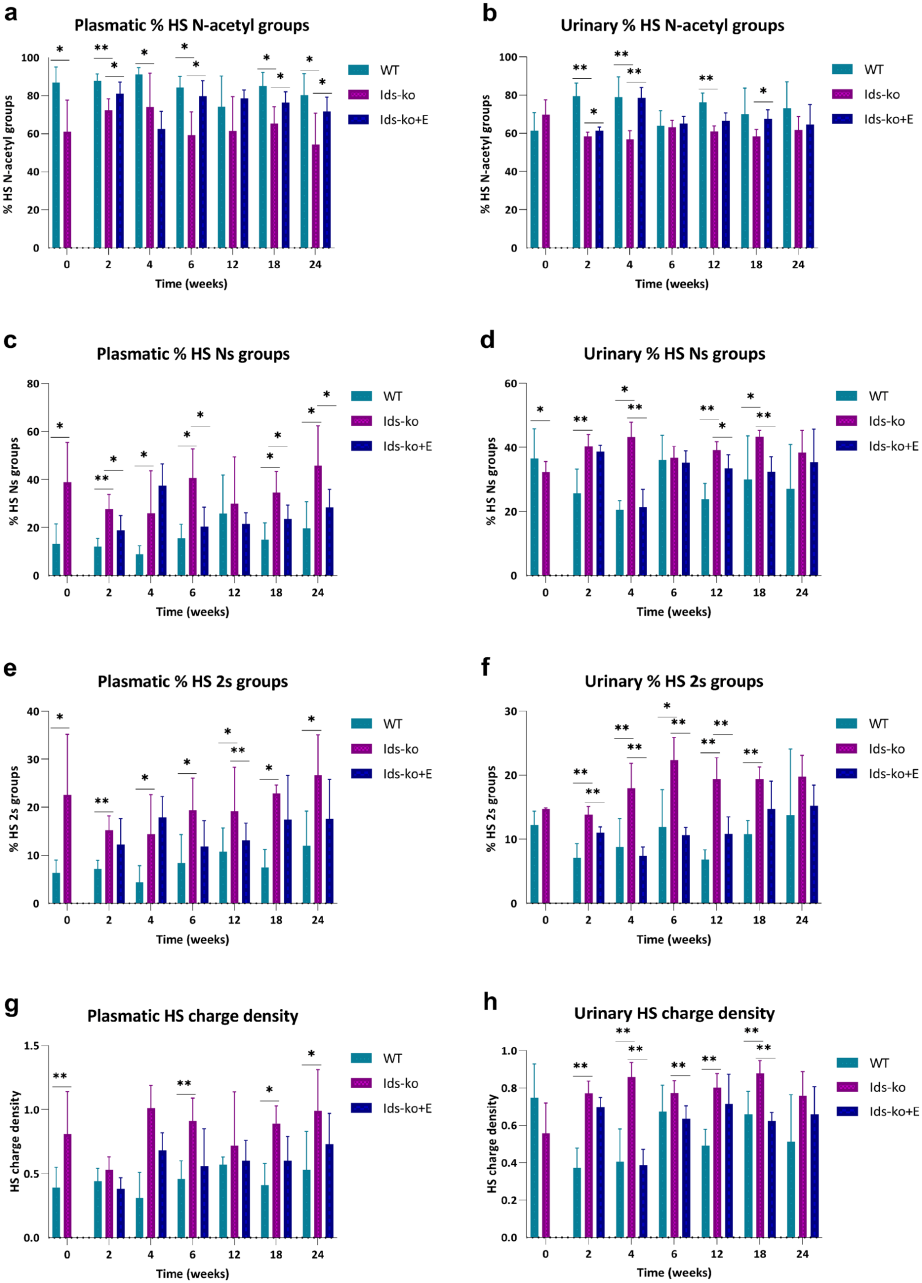
Structural analysis of HS in plasma and urine by CE-LIF. Percentages of *N*-acetyl (**a**, **b**), *N*-sulfo (**c**, **d**), 2-sulfo groups (**e**, **f**), and charge density (**g**, **h**) of HS, measured in plasma and urine of WT, Ids-ko, and ERT-treated Ids-ko (Ids-ko + E) mice, during a time course of 24 weeks at *T* = 0, 2, 4, 6, 12, 18, and 24 weeks. All values are represented as mean ± SD. *n* = 6–7 per group. Asterisks indicate a statistically significant difference between Ids-ko and WT and between Ids-ko and Ids-ko + E at the same time point (Mann–Whitney *U* test; * 0.01 ≤ *p*-value < 0.05; ** 0.001 ≤ *p*-value < 0.01; *** *p*-value < 0.001)

Analogously to CS + DS, HS structural analysis allowed the quantitation of 8 species with a different pattern of sulfation/acetylation (Table 1). Figure 4 shows the percentage of *N*-acetyl, *N*-sulfo, 2-sulfo groups and whole charge density of HS measured in plasma and urine of untreated and ERT-treated Ids-ko, and of WT mice.

The comparison Ids-ko vs WT revealed significant differences in all species at almost all time points in plasma (with exception of *T* = 12 weeks for *N*-acetyl and *N*-sulfo groups). In urine, the same comparison evidenced significant variations at 2, 4, and 12 weeks for the *N*-acetyl, at all time points except 6 and 24 weeks for *N*-sulfo species, and at 2, 4, 12, and 18 weeks for HS 2s (Fig. 4a–f). Considering the effect of treatment, this was more evident in plasma for *N*-acetyl and *N*-sulfo HS with a significant drop at 2, 6, 18, and 24 weeks for both types of disaccharides, while 2s species decrease was more considerable in urine, with statistical significance after 2, 4, 6 and 12 weeks of treatment (Fig. 4a–f). Consequently, these changes of concentration of the different sulfated/acetylated forms of disaccharides determined a variation of the whole charge density of HS with no relevant differences between plasma and urine (Fig. 4g, h). A statistically significant growth of HS charge density in Ids-ko vs WT mice was observed at 0, 6, 18, and 24 weeks in plasma and at 2, 4, 12, and 18 weeks in urine. Following ERT, HS charge density slightly declined, tending towards WT values, with a statistical significance only in urine, at 4, 6, and 18 weeks.

As regarding HS 6s, statistically significant variations were observed mostly in urine at 2 and 4 weeks of follow-up for both types of comparisons (Ids-ko vs WT, and treated vs untreated Ids-ko) (Online Resource 1–Fig. S3).

## Discussion

Main aim of the present study was to quantify and structurally characterize the plasma and urine GAG composition in Ids-ko mice compared to WT animals and to evaluate their progressive changes along 24 weeks of treatment with hr-IDS, compared to untreated animals. At the best of our knowledge, this is the first report of a detailed structural GAG analysis applied to a 24-week ERT follow-up of the mouse model of MPS II. According to the enzyme defect, the lack of functional IDS in MPS II does not permit the cleavage of chemical sequences in which the sulfate groups are located in the C2 position of terminal non-reducing end of IdoA residues in HS and DS causing their accumulation in tissues and organs [42].

One of the most common, simple, and non-invasive analytical approach for total GAG quantitation is the colorimetric assay by DMB [43], able to measure total urine GAG levels, useful for an early diagnosis [44], or to follow the effectiveness of the available therapies [31, 32]. In this study, this technique, applied as first level analysis in urine, evidenced a clear and substantial reduction of total GAGs from 2 weeks of treatment until the end of the follow-up, in mice treated with ERT. Notably, although in the last years the DMB method for GAG quantitation has been progressively replaced by more advanced techniques based on mass spectrometry, it is still being used as first level analysis for diagnostic purposes, given its cheapness and ease of execution. However, attention should be paid in the diagnosis of patients with haematuria and proteinuria as blood and hemoglobin may interfere with the analysis [45].

A more specific analytical procedure is CE-LIF, an approach that can potentially provide absolute GAG species quantitation independently from calibration curves, age of patients, or creatinine levels for urine.

Moreover, CE-LIF, due to its high resolving power, sensitivity, and high-throughput capacity, is useful in the analysis of complex and simple carbohydrates. It permits the analysis of many samples in about 48–50 h at relatively low costs. Finally, along with modifications of total as well as single GAG content, this analysis offers a method able to measure changes in GAG composition and structure with the definition of candidate biomarkers as those highlighted in the present study.

A similar analytical procedure was previously adopted in a large cohort of healthy newborns of 2–3 days of age and in a small group of normal adult subjects, to obtain a basic profile of total content of urine GAGs, their composition, and structural signatures [39]. A comparable analysis was also performed in dried blood spots obtained from 600 healthy newborns and from a small group of MPS subjects matched for age, confirming that the plasma modifications of GAG composition and structure would be useful for a possible early diagnosis of various MPS subtypes [46].

Consistently with the enzyme deficit, in this study, we observed in the Ids-ko mice a statistically significant increase of HS and CS + DS levels in urine at all time points considered, while in plasma, this variation resulted less marked. In addition, the enzyme treatment allowed a drastic reduction of HS and a considerable decline of galactosaminoglycans in urine, while in plasma, these changes were clear only at a few time points for both types of GAGs. A reduction of both CS + DS and HS is also reported by Menkovic et al. [47] by UPLC–MS/MS method in urine and plasma collected during a 16-week follow-up of ERT-treated and untreated Ids-ko mice.

We also observed in Ids-ko mice a moderate increase in both biological fluids of the non-sulfated HA, as already observed in several organs of an MPS IIIA mouse model [48], possibly accumulated through a secondary storage mechanism.

The relative proportion of the main sulfated GAGs, galactosaminoglycans (CS + DS) and glycosaminoglycans (HS), is altered in both plasma and urine of Ids-ko animals compared to WT. Indeed, both DS and HS increase in ko animals due to the lack of IDS, but the percentage of increase of HS is higher than that of DS; thus, the galactosaminoglycans/HS ratio decreases. As this ratio is an absolute value calculated through the absolute content of sulfated GAGs and it is restored to physiological values in both biological fluids after hr-IDS treatment, we can likely consider it as a potential biomarker of MPS II.

The determination of the disaccharide pattern by the present analytical procedure also permits the evaluation of the sulfation level of the different GAGs and of the overall charge density. The IDS deficit causes an overall increase of the sulfation level, due to the undegraded HS and DS, accumulated in the different organs and tissues and released in the biological fluids. This effect on the variation of the sulfation status, depending on the pathological conditions and on the ERT treatment, was registered as similar in blood and urine.

Among the differentially sulfated CS-DS disaccharides analyzed, also the percentage of the 2,4dis (disaccharide B) could be reasonably considered a candidate biomarker for MPS II. Indeed, it is substantially increased in Ids-ko mice in both body fluids, and it tends to considerably decline after ERT, especially in plasma (at all time points except one), but also in urine (at three time points). Notable alterations of the disaccharide B concentrations were previously registered in biological fluids of patients affected by Hurler-Scheie syndrome (attenuated MPS I) [49].

In healthy conditions, besides DS, IDS also cleaves the sulfate group linked in C2 position of the IdoA residues in HS. Consequently, as expected, a detailed disaccharide mapping showed in the Ids-ko mouse a deep modification of HS structure in both plasma and urine. Among the different HS species quantified, the change in the percentage of disaccharides sulfated in position 2 of IdoA (HS 2s) was the most significant in the comparison of both untreated vs WT and treated vs untreated, as HS 2s is the main product of the failed cleavage of the sulfate group in position C2. Consequently, this species could be reasonably added to the list of potential biomarkers of disease and of therapeutic efficacy for MPS II. The variations of *N*-acetyl and *N*-sulfo forms of HS are also noteworthy, especially in plasma, although to a lower extent.

Already after 2 weeks of infusion, ERT was effective in rapidly reducing blood and urine GAG content, and to partially restore some chemical features, to levels that remained rather steady during the entire 24 weeks of treatment. Despite this strong reduction, we never observed a complete normalization, in particular of some structural parameters, of HS and DS. This is consistent with what was previously observed following ERT administration in MPS patients. Fujitsuka et al. evidenced in the blood of MPS II patients, treated with ERT for at least 1 month, a decrease of DS, HS0S, and HSNS, but without statistical significance [50]. In two MPS I patients, DS plasma reduction was observed up to ~ 80–85% of the initial concentration during the first 10-month treatment period; however, ERT was unable to totally remove DS from the blood [49]. The inability of ERT to normalize GAGs further supports the presence of pathogenetic mechanisms other than the primary GAG storage, and the consequent impairment of autophagic process, contributing to the complex clinical manifestations of MPSs [51]. Among them, the alteration of different signaling pathways, like fibroblast growth factor and sonic hedgehog, has been reported [8, 9] as well as the impairment of ion homeostasis and of other intra- and extracellular processes [15, 51]. In conclusion, the illustrated analytical procedure was able to measure changes in plasma and urine GAG concentration, composition, and chemical structure due to the enzyme defect in the MPS II mouse model, which could be significantly reduced with enzyme treatment. These modifications are therefore potentially useful for precocious diagnosis and as markers of therapeutic efficacy.

## Supplementary Information

Below is the link to the electronic supplementary material.

Online Resource 1 Supplementary file1 (PDF 909 KB)

## Data Availability

The datasets generated and analyzed during the current study are available from the corresponding author Volpi N. on reasonable request.
