# Supplementary material for: Glycosaminoglycan signatures in body fluids of mucopolysaccharidosis type II mouse model under long-term enzyme replacement therapy
Source: J Mol Med (Berl). 2022 Jul 11;100(8):1169–79. doi: 10.1007/s00109-022-02221-3 (PMC9329393; doi:10.1007/s00109-022-02221-3)
Supplement: Supplementary file 1 — Online Resource 1 Supplementary file1 (PDF 909 KB) [file 109_2022_2221_MOESM1_ESM.pdf]

Online Resource 1

- **Figure S1**
- **Figure S2**
- **Figure S3**

Glycosaminoglycan signatures in body fluids of Mucopolysaccharidosis type II mouse model under long-term enzyme replacement therapy

Journal of Molecular Medicine
Authors: Maccari F, Rigon L, Mantovani V, Galeotti F, Salvalaio M, D'Avanzo F, Zanetti A, Capitani F, Gabrielli O, Tomanin R and Volpi N

Corresponding authors:
Nicola Volpi (e-mail address: volpi@unimo.it) - Department of Life Sciences, University of Modena and Reggio Emilia, Modena, Italy
Rosella Tomanin (e-mail address : rosella.tomanin@unipd.it)- Department of Women's and Children's Health, University of Padova, Italy

Online Resource 1 - Figure S1

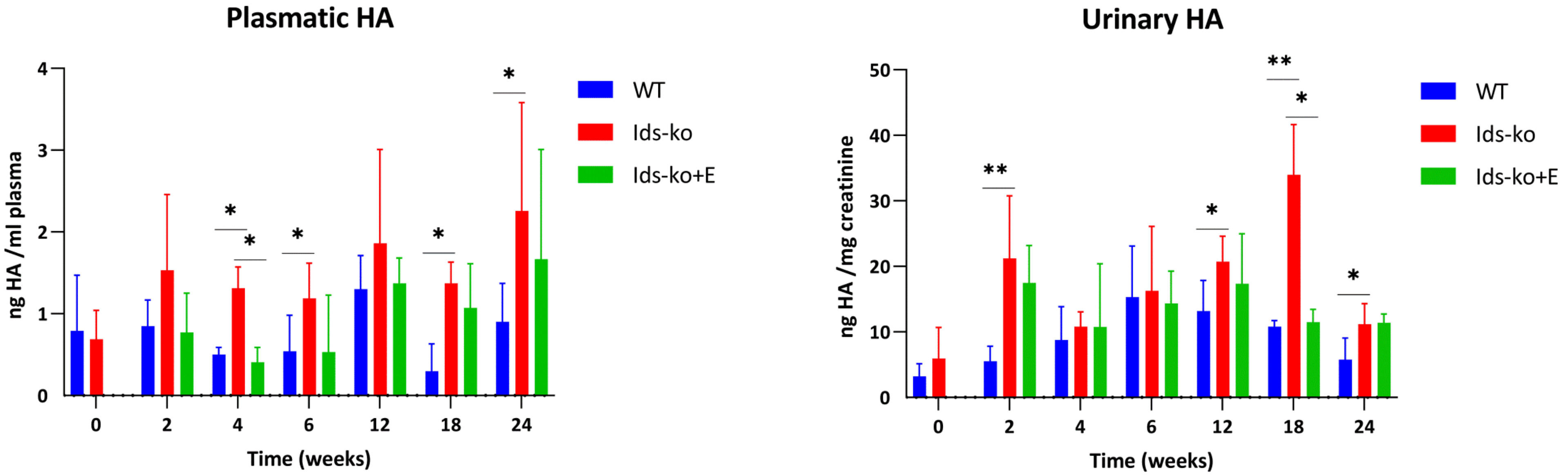

Figure S1. Quantitation of plasmatic and urinary HA by CE-LIF. HA concentration measured in plasma and urine of wild type, Ids-ko and Ids-ko mice treated with ERT (Ids-ko+E), during a time course of 24 weeks at T = 0, 2, 4, 6, 12, 18, 24 weeks. All values are represented as mean ±SD. n = 6-7 for each group. Asterisks indicate a statistically significant difference between Ids-ko and WT and between Ids-ko and Ids-ko+E at the same time point (Mann–Whitney U test; * $0.01 \le p\text{-value} < 0.05$; ** $0.001 \le p\text{-value} < 0.01$)

Online Resource 1 - Figure S2

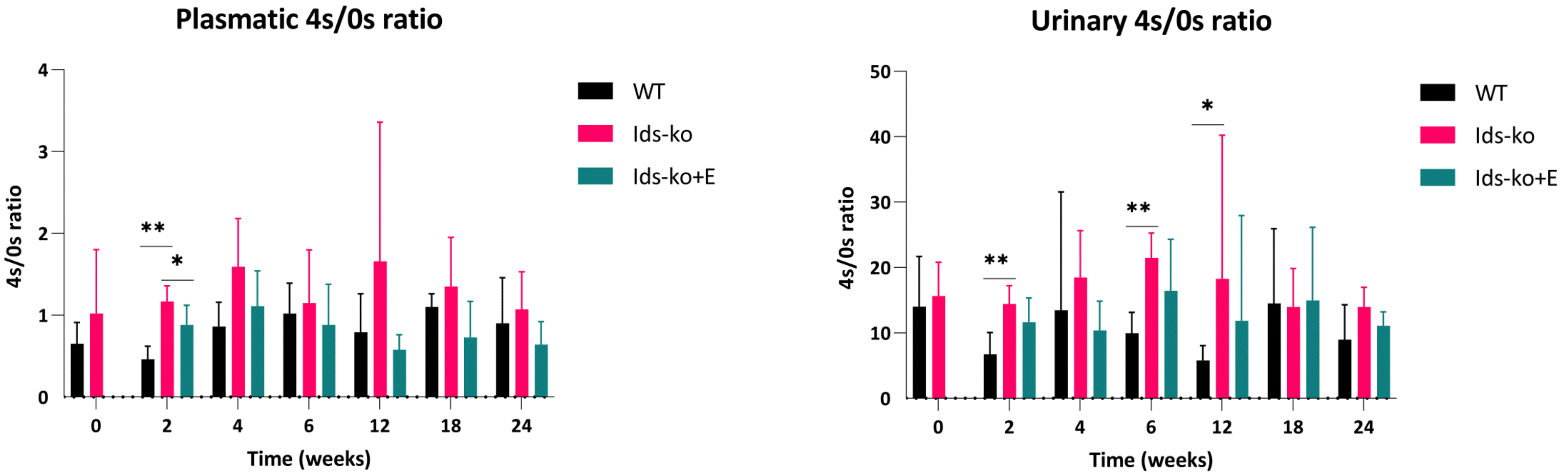

Figure S2. Plasmatic and urinary CS+DS 4s/0s disaccharides ratio. Ratio between the percentage of disaccharide 4s and the percentage of non-sulfated disaccharide (4s/0s) of CS+DS measured in plasma and urine of wild type, Ids-ko and Ids-ko mice treated with ERT (Ids-ko+E), during a time course of 24 weeks at T = 0, 2, 4, 6, 12, 18, 24 weeks. All values are represented as mean ±SD. n = 6-7 for each group. Asterisks indicate a statistically significant difference between Ids-ko and WT and between Ids-ko and Ids-ko+E at the same time point (Mann–Whitney U test; * 0.01≤p-value<0.05; ** 0.001≤p-value<0.01).

Online Resource 1 - Figure S3

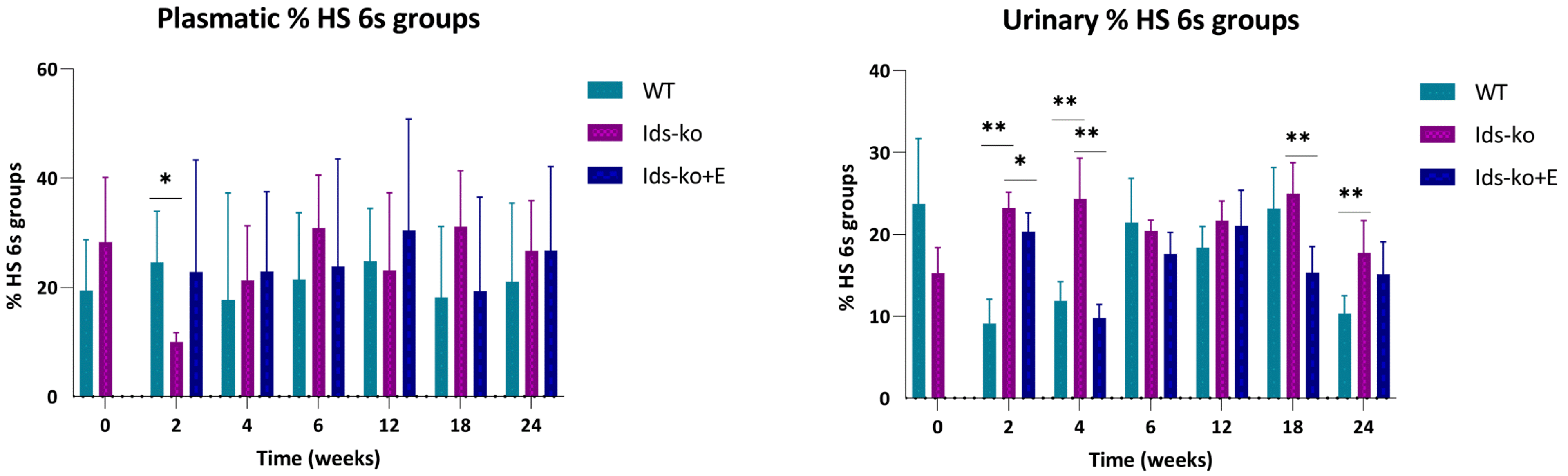

Figure S3. Percentage of plasmatic and urinary HS 6s disaccharides. Percentage of HS 6-sulfated disacaccharides measured in plasma and urine of wild type, Ids-ko and Ids-ko mice treated with ERT (Ids-ko+E), during a time course of 24 weeks at T = 0, 2, 4, 6, 12, 18, 24 weeks. All values are represented as mean ±SD. n=6-7 for each group. Asterisks indicate a statistically significant difference between Ids-ko and WT and between Ids-ko and Ids-ko+E at the same time point (Mann–Whitney U test; * 0.01≤p-value<0.05; ** 0.001≤p-value<0.01).
